# On the taxonomic status and distribution of African species of *Otomops* (Chiroptera: Molossidae)

**DOI:** 10.7717/peerj.4864

**Published:** 2018-05-24

**Authors:** Bruce D. Patterson, Paul W. Webala, Michael Bartonjo, Julius Nziza, Carl W. Dick, Terrence C. Demos

**Affiliations:** 1 Integrative Research Center, Field Museum of Natural History, Chicago, IL, USA; 2 Department of Forestry and Wildlife Management, Maasai Mara University, Narok, Kenya; 3 Mammalogy Section, National Museums of Kenya, Nairobi, Kenya; 4 Regional Headquarters, Mountain Gorilla Veterinary Project, Musanze, Rwanda; 5 Department of Biology, Western Kentucky University, Bowling Green, KY, USA

**Keywords:** Sympatry, Speciation, Range extension, Taxonomy, East Africa, Ectoparasites, Echolocation

## Abstract

**Background:**

Free-tailed bats of the genus *Otomops* are poorly known, and most species are documented from a handful of widely scattered localities. Recently, two allopatric species of *Otomops* were recognized in continental Africa: *Otomops martiensseni* (Matschie, 1897) in southern, central and western Africa, and the new species *O. harrisoni* Ralph et al., 2015 in the northeast and in Yemen.

**Methods:**

We collected additional samples of *Otomops* in Kenya and Rwanda where the ranges of these taxa approach one another to clarify their geographic ranges and taxonomic status. Mitochondrial and nuclear intron sequences served to identify and delimit species; we also documented their echolocation call variation and ectoparasite complements.

**Results:**

*Otomops martiensseni*, the southern African species, was documented in northern Kenya in Marsabit National Park. *O. harrisoni*, the northeastern African–Arabian species, was documented in southern Kenya and in a cave in Musanze District, Rwanda. Moreover, individuals of both species were found together at the Musanze cave, establishing them in precise spatial and temporal sympatry. Analyses of mitochondrial and nuclear loci identify no evidence of admixture between these forms, although available samples limit the power of this analysis. Echolocation call differences are also apparent among the three localities we analyzed. Three orders of insects and two families of mites are newly reported as ectoparasites of *O. harrisoni.*

**Discussion:**

Our results corroborate species rank for *O. harrisoni* and establish a zone of potential geographic overlap with *O. martiensseni* spanning at least 800 km of latitude. The new records establish the species in sympatry in northern Rwanda and add an additional species to the bat faunas of both Kenya and Rwanda. Future studies are needed to understand *Otomops* roosting requirements and movements, thereby explaining the paucity of known colonies and yielding better estimates of their conservation status. The discovery of mixed roosting associations in Rwanda invites further investigation.

## Introduction

Big-eared free-tailed bats of the genus *Otomops* Thomas, 1913 have a paleotropical distribution, ranging from southern Africa and Madagascar to the Arabian Peninsula, India, Indonesia, and New Guinea. Most species of *Otomops* are poorly known and are documented at a small number of widely scattered localities. Five of the eight species recognized by the IUCN are listed as Data Deficient, and only the Malagasy species *O. madagascariensis* Dorst, 1953 is listed as being of Least Concern (http://www.iucnredlist.org/).

For most of the past century, all *Otomops* from Africa and Madagascar were recognized as *O. martiensseni* (Matschie, 1897), with a type locality at Magrotto Plantation, SE Usambara Mountains, Tanzania. Hayman & Hill (1971) further distinguished southern African populations as *O. m. icarus* Chubb, 1917 and those from Madagascar as *O. m. madagascariensis*. However, Peterson, Eger & Mitchell (1995) accorded specific rank to *O. madagascariensis.* More recent revisionary works (Lamb et al., 2008; Ralph et al., 2015) have synonymized *O. m. icarus* with *O. martiensseni* and have additionally shown that populations from northeastern Africa and the Arabian Peninsula are distinct and deserve recognition as a distinct species, *O. harrisoni* Ralph et al., 2015.

Building on earlier studies (Lamb et al., 2006, 2008), Ralph et al. (2015) recovered *O. martiensseni* (including *icarus*) and *O. harrisoni* each as reciprocally monophyletic clades in analyses of mitochondrial DNA sequences (cytochrome-*b* (cyt-*b*) and control region). However, divergence between the two taxa in these highly variable loci was found to be modest, averaging 2.1%. Analyses of nuclear intron sequences and microsatellite variation (Ralph et al., 2015) also supported the separation of northern and southern African *Otomops* into two distinct clusters, again with very modest divergence and limited interpopulational variation. Morphometric analyses of cranial and dental variation showed the continental African *Otomops* clustered into distinct but overlapping groups. Quantitative morphometrics and reciprocal monophyly of mitochondrial lineages were used to diagnose *O. harrisoni* as a new, distinct species (Ralph et al., 2015). Ecological niche modeling, bacular variation, and qualitative characters were also presented to characterize their newly named species and differentiate it from *O. martiensseni*.

The name *O. martiensseni* is based on a late-19th century holotype (MNHB 97523), which could be used in morphological analyses but not genetic ones. Ralph et al. (2015) used quantitative morphology to ally the holotype with southern African *Otomops*. Both genetic and morphometric analyses of another northern Tanzanian specimen assigned it—and by extension the name *O. martiensseni—*to the southern African form. As mapped by Ralph et al. (2015), the two species have non-overlapping distributions: *O. harrisoni* in northeastern Africa (Kenya, Ethiopia, Eritrea, and Djibouti) and Yemen, and *O. martiensseni* in Ivory Coast, Central African Republic, Democratic Republic of Congo, Uganda, Tanzania, and points south. Nowhere were the two taxa found to be sympatric.

**Figure 1:**
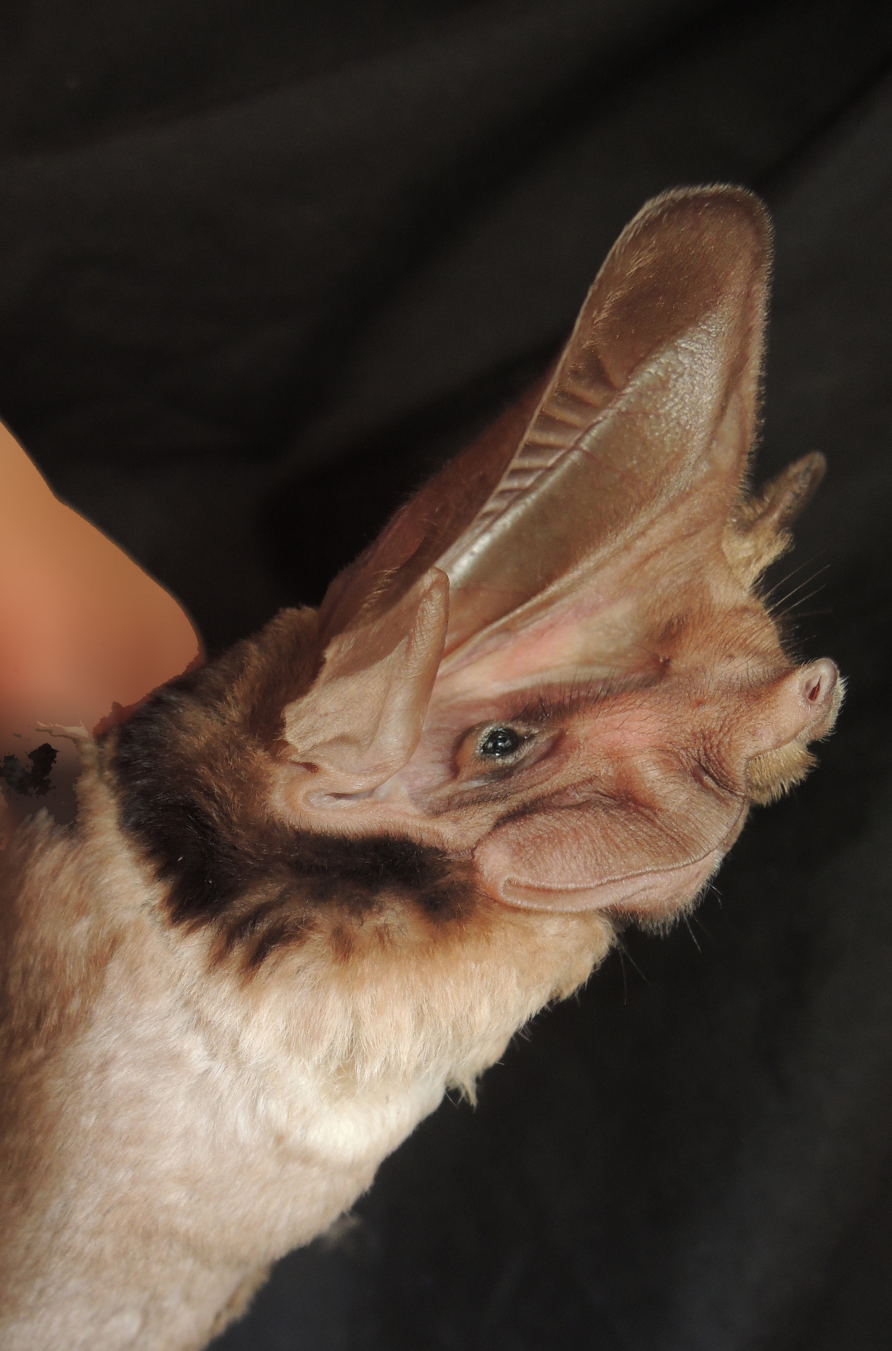
Photo of an *Otomops martiensseni* (NMK 184401) from Marsabit National Park. *Otomops martiensseni* (NMK 184401) from Marsabit National Park, Marsabit County, Kenya (photo by B. D. Patterson).

**Figure 2:**
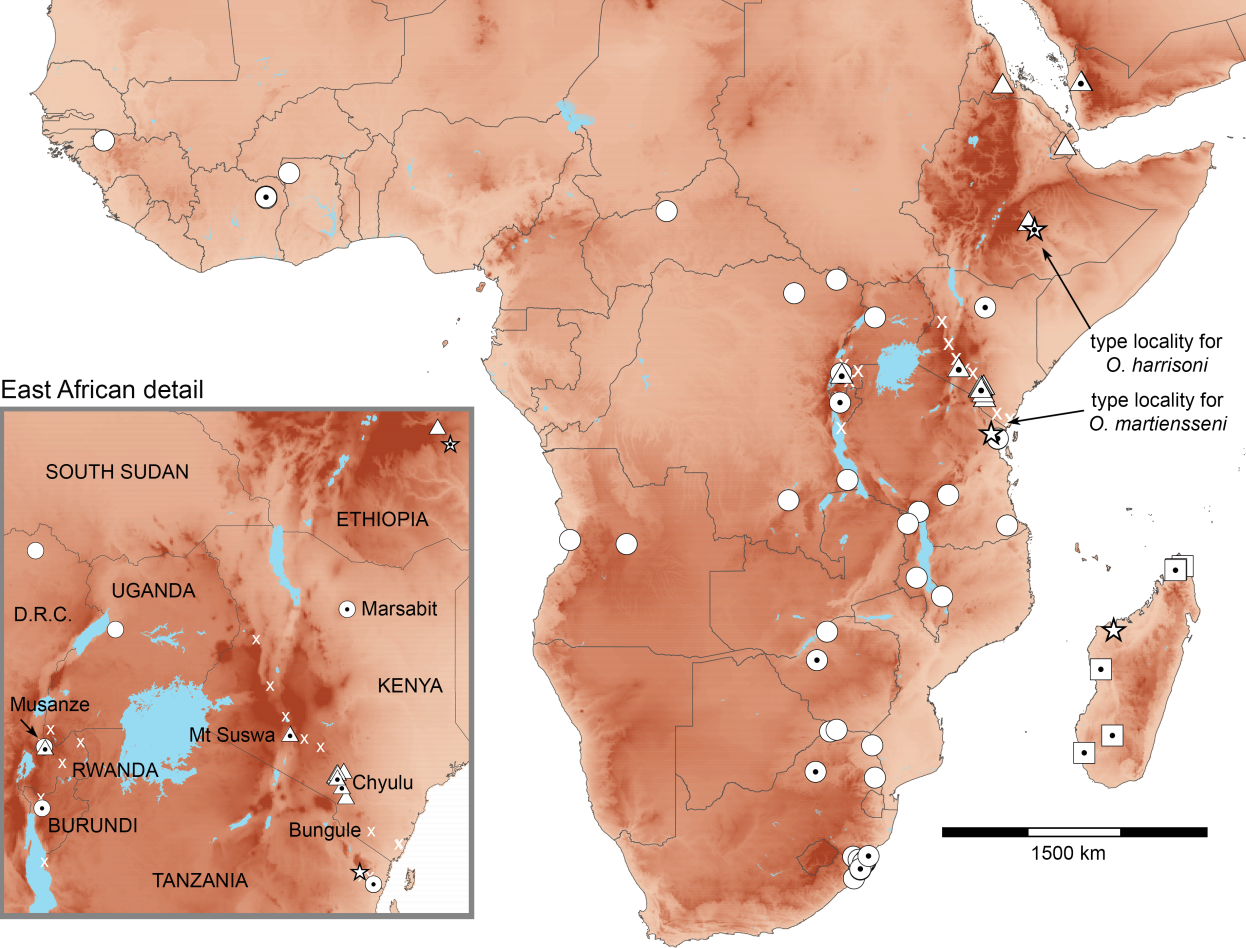
Distributions of African *Otomops* projected on topography. Triangles identify *O. harrisoni* records, circles those of *O. martiensseni*, and squares those of *O. madagascariensis*. Dotted symbols indicate identifications that have been confirmed by genetic analysis. Stars indicate type localities. X identifies *Otomops* records in the region of range overlap—most published as *O. martiensseni*—whose identification need further confirmation.

We recently completed a multi-year survey of bat populations across Kenya, encountering *Otomops* in three principal locations. These included Mount Suswa Conservancy, Caves #14C and #18A in Kajiado County; Chyulu Hills National Park, Guano cave 3 in Makueni County; and Marsabit National Park and Reserve, 1.3 km SE campground and headquarters in Marsabit County. The Suswa and Chyulu populations were sampled from their lava-tube day roosts; the Marsabit record was taken at 7:15 pm in the top shelves of a triple-high net system (https://batmanagement.com/) some 6 m above ground over a road through second-growth forest (Figs. 1 and 2). In addition, PWW and JN captured nine *Otomops* (species undetermined) from a single volcanic tunnel, Musanze Tourist Cave in Musanze District, northern Rwanda, releasing and recording vocalizations from six and preserving three as vouchers. These additional samples from localities near the presumed contact zone of *O. martiensseni* and *O. harrisoni* enable further assessments of their taxonomic status and respective geographic distributions.

## Materials and Methods

### Sampling

The majority of new nucleotide sequences generated for this study (from 24 individuals) were from voucher specimens collected during fieldwork in Kenya and Rwanda (Supplemental Material). Field methods were approved by Field Museum of Natural History’s IACUC (2012-003). Kenyan work was permitted by Kenya Wildlife Service, KWS/4001 and the Kenya Forest Service, RESEA/1/KFS/75). Rwandan fieldwork was carried out with authorization by the Rwanda Development Board. Tissue samples were taken at the time of capture when the vouchers were also screened for ectoparasites. An additional 30 cyt-*b* sequences of *Otomops* sampled from across Africa and Asia were downloaded from GenBank, mostly from reports by Ralph, Lamb, and their associates (Lamb et al., 2006, 2008, 2011; Ralph et al., 2015) (Supplemental Information 1). We generated sequences for nuclear introns ACPT and ACOX2 from 22 recently collected individuals to evaluate the congruence of mitochondrial and nuclear sequences. Based on the phylogeny of Lamb et al. (2011), *O. wroughtoni* of tropical Asia was used as an outgroup for the mitochondrial analyses of African and Malagasy samples, and *O. madagascariensis* was used in analyses of African *Otomops* involving the nuclear introns. To map species distributions, we supplemented our genetic records with literature and museum records (Supplemental Information 2). Because discrete characters diagnostic of *O. harrisoni* and *O. martiensseni* are lacking, and quantitative morphological analyses show overlapping distributions (Ralph et al., 2015), we based our identifications on molecular analyses which unequivocally separate these forms.

### DNA extraction, amplification, and sequencing

Whole genomic DNA was extracted from tissue samples using the Wizard SV 96 Genomic DNA Purification System (Promega Corporation, Fitchburg, WI, USA). Specimens were sequenced for mitochondrial cyt-*b* using the primer pair LGL 765F and LGL 766R that amplify the entire cyt-*b* gene (Bickham et al., 2004; Bickham, Wood & Patton, 1995) and two unlinked autosomal nuclear introns; ACPT (intron 4) and ACOX2 (intron 3), using primers described in Salicini, Ibáñez & Juste (2011). PCR amplifications were carried out in 25 μl reaction volumes as follows: 1–2 μl of template DNA (approx. 5–25 ng), 12.5 μl of One*Taq* 2× Master Mix with Standard Buffer (New England BioLabs Inc., Ipswich, MA, USA), and 1 μl of 10 μM forward and reverse primers. Thermal conditions for the cyt-*b* gene consisted of an initial denaturation step at 95 °C for 3 min, followed by 38 cycles consisting of 45 s at 95 °C, 30 s at 50 °C, 2.5 min at 70 °C, followed by a final extension step of 5 min at 70 °C as in Trujillo et al. (2009). Thermal conditions for the two nuclear loci consisted of an initial denaturing step at 95 °C for 3 min; one cycle of 95 °C for 15 s, 65 °C for 30 s, 72 °C for 1 min; followed by one cycle each at annealing temperature in 1 °C decrements from 65 °C (64–56 °C); 32 cycles of 95 °C for 15 s, 55 °C for 30 s, 72 °C for 1 min; followed by a final extension step of 5 min at 72 °C as in Dool et al. (2016). Amplified products were purified using ExoSAP-IT (Thermo Scientific, Waltham, MA, USA). Sequencing was carried out in both directions on an ABI 3100 thermocycler (Applied Biosystems, Foster City, CA, USA) at the Pritzker Laboratory for Molecular Systematics and Evolution (FMNH). Chromatographs were checked manually, assembled and edited using GENEIOUS PRO 11.04 (Biomatters Ltd.). Sequences from each locus were aligned independently using the MUSCLE algorithm (Edgar, 2004) with default settings in GENEIOUS. Sequence data from cyt-*b* were translated into amino acids and inspected for deletions, insertions, and premature stop codons to exclude paralogous sequences. Alignments for all data sets were inspected visually and determined to be unambiguous. Newly generated sequences were deposited in GenBank (MH010730–MH010797; Table S1, Supplemental Information).

### Molecular methods

The best supported models of nucleotide substitution for cyt-*b* and the two nuclear introns were determined using the Bayesian Information Criterion (BIC) on the maximum likelihood topology estimated for each model independently in jMODELTEST2 (Darriba et al., 2012) on the CIPRES Science Gateway v.3.3 (Miller, Pfeiffer & Schwartz, 2010). We calculated interspecific uncorrected sequence divergences (*p-*distances) for cyt*-b* in MEGA v.7.0.26 (Kumar, Stecher & Tamura, 2016).

Maximum likelihood estimates of gene trees for cyt-*b* and a concatenated alignment of cyt-*b*, ACOX2, and ACPT were made using the program RAxML-HPC v.8 (Stamatakis, 2014) on the CIPRES portal (alignments in Supplemental Information 3 and 4). The alignments for the concatenated phylogenetic analyses included 22 *Otomops* individuals newly sequenced for this study. An additional 30 *Otomops* cyt-*b* sequences obtained from GenBank were added to individual cyt-*b* phylogenetic analyses in order to place our newly sequenced individuals in a broader phylogenetic context and incorporate the results of previous studies. We conducted analyses using the rapid bootstrapping algorithm and search for best-scoring ML tree algorithm under the GTRGAMMA model with 1,000 bootstrap replicates. Bayesian gene tree analyses used MrBayes v.3.2.6 (Ronquist et al., 2012) on the CIPRES portal to infer gene trees for cyt-*b* and a partitioned concatenated alignment of cyt-*b*, ACOX2, and ACPT. Two replicates were run to ensure proper mixing had occurred. Nucleotide substitution models were unlinked across partitions and were allowed to evolve at individual rates in the concatenated alignment. Four Markov chains with default heating values were conducted for 10^7^ generations and sampled every 1,000th generation. Stationarity was assessed using trace plots and ESS values >200 in Tracer v.1.6 (Rambaut et al., 2014). The first 2,500 samples were discarded as burn-in and the remaining 7,500 samples formed the posterior probability (PP) distributions. Majority-rule consensus trees with estimates of Bayesian support were generated for each analysis. Gene trees were visualized using FigTree v. 1.4.3 (Rambaut, 2016).

### Echolocation call recordings and analysis

We recorded echolocation calls in real time from individual *Otomops* hand-released outside cave entrances using a handheld ultrasound detector (Petterson D1000X; Pettersson Elektronik AB, Uppsala, Sweden; 384 kHz sampling rate, 16 bit resolution). After release, African big-eared bats use frequency-modulated sweep calls and gradually come back to normal search-phase mode when flying high above ground (Fenton et al., 2002). Pulses used in analysis were selected once the sequences lacked this gradual change during the search-phase mode (Britzke et al., 2011). We were unable to record calls from the lone capture from Marsabit, Kenya, or from any genetically sampled bats, which were preserved as museum vouchers.

For sound analysis, a customized 512-point fast Fourier transform (FFT) was used with a Hanning window for both spectrograms and power spectrum. Following Jung, Molinari & Kalko (2014), we characterized echolocation calls by measuring peak frequency or frequency with maximum energy, maximum frequency (StartF), minimum frequency (EndF), and duration of calls (Dur) using Kaleidoscope v.3.1.4b (Wildlife Acoustics, Maynard, MA, USA). Ten calls with the best signal-to-noise ratios were measured for each of 16 individual bats (Supplemental Information 5). Analyses of variance and multiple comparisons tests were based on log-transformed variables.

## Results

Individual alignments consisted of 1,140 nucleotides for cyt-*b* (*n* = 54, including 30 GenBank sequences), and 489 and 425 nucleotides for ACOX2 (*n* = 22) and ACPT (*n* = 22), respectively. jModeltest2 analyses identified the HKY + G substitution model as best supported for the 41 individual cyt-*b* alignment, HKY for the 22 individual cyt-*b* and ACPT alignments, and K80 for the 22 individual ACOX2 alignment. Concatenation of one mitochondrial locus and two introns resulted in an alignment of 2,035 nucleotides, with no missing data, from 22 individuals. The concatenated alignment of all three loci consisted of 72 variable sites and 30 parsimony informative sites.

The average interspecific genetic distance (uncorrected cyt-*b p*-distance) between *O. harrisoni* (*n* = 20) and *O. martiensseni* (*n* = 3) individuals newly sequenced for this study was 2.5%. Intraspecific genetic distances were low (0.10% within *O. harrisoni* and 0.30% within *O. martiensseni*).

*Otomops martiensseni* and *O. harrisoni* are each recovered as reciprocally monophyletic lineages by both Bayesian Inference and ML phylogenetic methods. Individual gene trees and the concatenated dataset also support these species as sisters in the concatenated phylogenetic analyses: PP = 1.0, bootstrap proportion (BP) = 100. This relationship is moderately supported in the cyt-*b* gene tree (PP = 0.80, BP = 72). Phylogenetic analyses of cyt-*b* that include four *Otomops* species strongly support the *O. harrisoni* + *O. martiensseni* clade as sister to *O. madagascariensis* (PP = 1.0, BP = 100).

**Figure 3:**
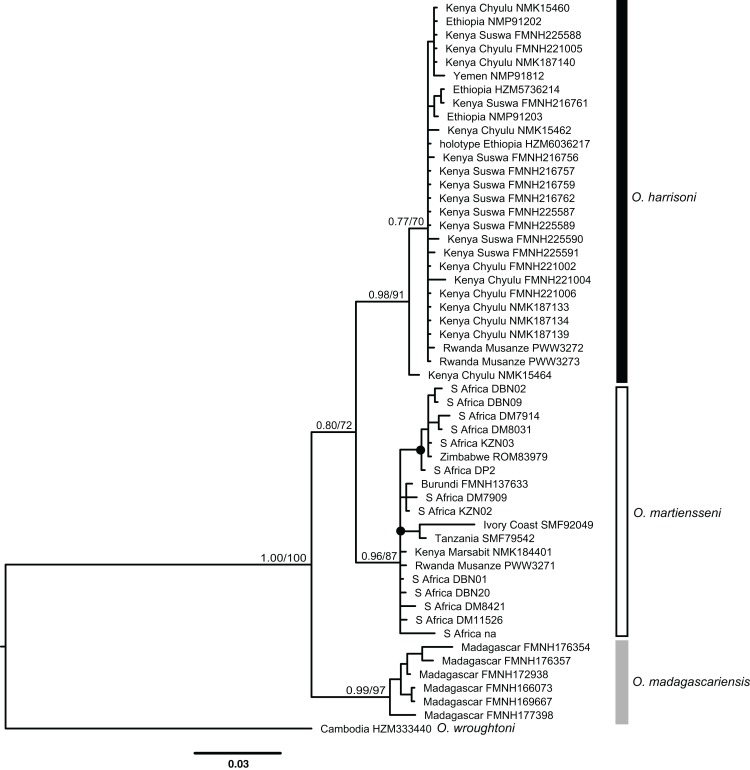
Bayesian cytochrome-*b* gene tree inferred using MrBayes. Numbers above branches of major nodes indicate Bayesian posterior probabilities followed by bootstrap values for maximum likelihood inferred using RaxML. Posterior probabilities ≥95% and bootstrap values ≥70% are denoted by filled circles on minor nodes.

**Figure 4:**
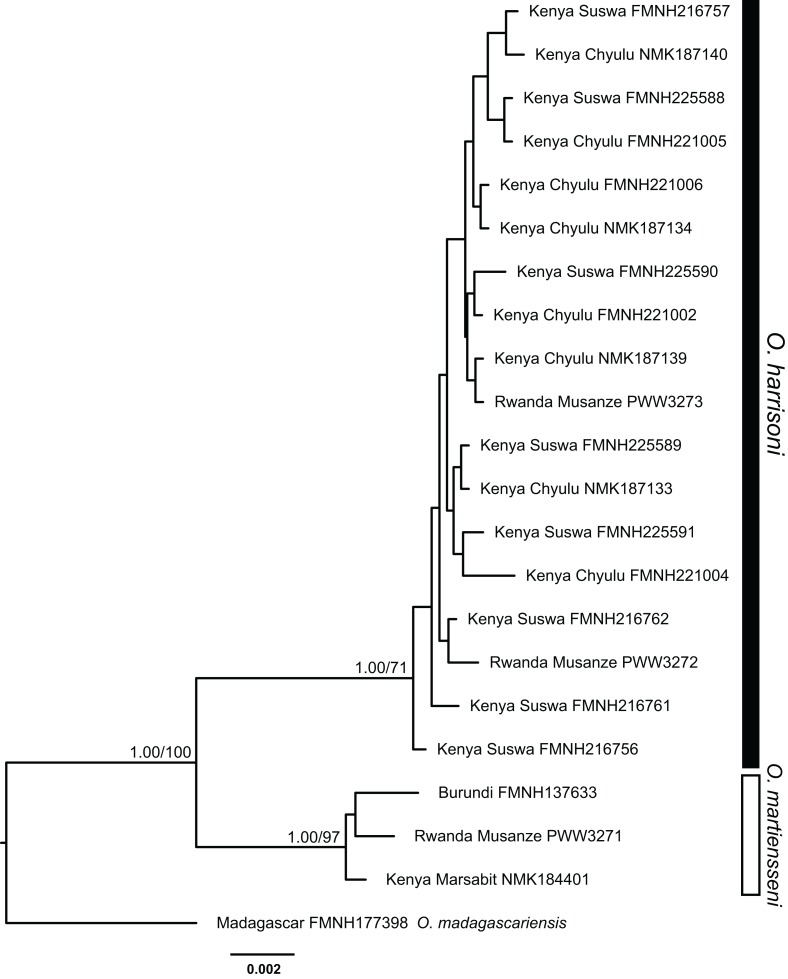
Bayesian phylogeny inferred from partitioned analysis of combined cytochrome-*b*, ACOX2, and ACPT sequences. Bayesian posterior probabilities followed by bootstrap values for maximum likelihood are indicated above branches of major nodes. Posterior probabilities ≥95% and bootstrap values ≥70% are denoted by filled circles on minor nodes.

Individuals within the *O. harrisoni* and *O. martiensseni* clades do not cluster by geography in any of the phylogenetic analyses and so do not exhibit appreciable geographic population structure. Remarkably, two individuals from Musanze Cave in Rwanda were recovered as members of the *O. harrisoni* clade, whereas the third individual sampled from that cave grouped with *O. martiensseni*. In addition, the individual from Marsabit, Kenya grouped within the *O. martiensseni* lineage (Figs. 3 and 4). Both mitochondrial and nuclear loci varied in parallel, supporting these species assignments.

The echolocation peak frequency of big-eared free-tailed bats (Table 1) differed significantly among cave sites (*F*_2,13_ = 12.583; *P* = 0.001), with *Otomops* from the Chyulu Hills (where only *O. harrisoni* were genotyped) echolocating at higher frequencies than those at either Suswa (where only *O. harrisoni* has been recorded; *P* = 0.002) or *Otomops* sp. from Musanze, where both species are documented (*P* = 0.001); no differences were evident between the latter two sites (*P* = 0.906). Both maximum (*F*_2,13_ = 8.327; *P* = 0.005) and minimum frequencies (*F*_2,13_ = 5.037; *P* = 0.024) differed significantly between sites. Maximum frequencies did not differ between samples from Mt Suswa and Chyulu Hills (*P* > 0.05), but Chyulu bats vocalized higher than those from Musanze (*P* < 0.01). Similarly, minimum frequencies did not differ between Mt Suswa and Chyulu Hills (*P* > 0.05), but again, Chyulu bats differed significantly from those at Musanze (*P* = 0.02). Call duration of echolocating bats from all sites did not significantly differ (*P* > 0.05).

**Table 1:** Search-phase call parameters for *Otomops* from Kenya and Rwanda. Means (±S.E.) are presented, with observed ranges in parentheses.

Echolocation call parameters	*Otomops harrisoni* Chyulu Hills, Kenya (*n* = 4)	*Otomops harrisoni* Mt Suswa, Kenya (*n* = 6)	*Otomops* sp. Musanze, Rwanda (*n* = 6)
Peak frequency (kHz)	17.18 ± 0.42 (16.40–17.95)	11.86 ± 0.72 (9.52–13.80)	11.48 ± 0.74 (9.44–13.49)
Maximum frequency (kHz)	21.67 ± 0.10 (21.48–21.90)	17.81 ± 1.70 (10.72–21.62)	13.21 ± 0.89 (10.33–15.89)
Minimum frequency (kHz)	12.53 ± 0.29 (11.80–13.13)	10.94 ± 0.47 (9.63–12.80)	10.34 ± 0.48 (9.15–11.31)
Call duration (ms)	10.72 ± 1.10 (7.57–12.67)	9.74 ± 1.21 (7.04–15.16)	8.41 ± 0.99 (5.03–11.34)

On 7 August 2011, we recovered at least five species of ectoparasite from bats inhabiting Tunnel 14C of the Mt Suswa roost, where only *O. harrisoni* has been documented (Supplemental Information 6). These include representatives of Diptera (Streblidae: *Brachytarsina alluaudi* (Falcoz), Hemiptera (Polyctenidae: *Hypoctenes clarus* Jordan), Siphonaptera (Ischnopsyllidae: *Lagaropsylla* sp.) and unidentified species of Acari belonging to the families Argasidae and Macronyssidae.

## Discussion

Additional sampling where the ranges of *O. harrisoni* and *O. martiensseni* approach one another supports the primary taxonomic conclusions of Ralph et al. (2015). Despite modest genetic differentiation in highly variable mitochondrial loci, the two taxa maintain genetic integrity and appear to function as species. Insofar as known, mitochondrial divergence patterns are repeated in unlinked nuclear loci, confirming species rank for the two continental African taxa.

The 2.5% average interspecific genetic distance (uncorrected cyt-*b p*-distance) we found between newly sequenced *O. harrisoni* (*n* = 20) and *O. martiensseni* (*n* = 3) individuals are comparable to earlier reports. With more extensive sampling of both *O. harrisoni* (*n* = 21) and *O. martiensseni* (*n* = 33), Ralph et al. (2015) calculated an average *p*-distance of 2.1%. This interspecific genetic differentiation is far lower than has been documented for other genera of Molossidae. For African free-tailed bats, Lamb et al. (2011) documented mean cyt-*b* genetic distances of 10.6% among four species of *Mops*, and a mean of 7.4% among seven species of *Chaerephon*. In the Neotropics, Moras et al. (2016) found that cyt-*b* divergence averaged 9.7% among eight species of the genus *Cynomops*, and López-Baucells et al. (2018) documented 12.1% mean sequence divergence among six species of *Eumops* in the mitochondrial COI locus. Differentiation between continental African *Otomops* species falls below the 5% threshold commonly found for species-level differences (Bradley & Baker, 2001), but this is also the case for many other bat genera (Velazco & Patterson, 2008, 2013) and even for small, terrestrial species such as shrews (Demos et al., 2016, 2017). Reciprocal monophyly of the two taxa despite more extensive sampling as well as parallel divergence in mitochondrial and nuclear loci support the species-level distinction of *O. harrisoni* and *O. martiensseni* drawn by Ralph et al. (2015).

The Mount Suswa and Chyulu populations of *O. harrisoni* have long been known and studied under the name *O. martiensseni* (Aggundey & Schlitter, 1984; Kayanja & Mutere, 1975; Kinoti, 1973). However, our Musanze records of *O. harrisoni* and the Marsabit record of *O. martiensseni* represent new country records for these species and establish new geographic range extensions for both species (Fig. 2). Relative to the distributions of these species depicted in Ralph et al. (2015), the Marsabit record extends the range of *O. martiensseni* 800 km north of confirmed records in Tanzania, nearly to Kenya’s Ethiopian frontier. On the other hand, the Musanze records of *O. harrisoni* lie 750 and 900 km to the west-southwest of the Suswa and Chyulu populations, spanning most of northwestern Tanzania. Additions to the bat faunas of both countries are hardly surprising, as East Africa supports several of Africa’s richest bat faunas (Herkt et al., 2016; Patterson & Webala, 2012).

The fact that the ranges of both species include most of Kenya calls into question the identity of various historical records of *Otomops*, particularly those lying far from genetically confirmed records, including Kwale, Lake Baringo, Nairobi, Naivasha, and Wei-Wei River, Kenya (Aggundey & Schlitter, 1984). To aid future workers, we have distinguished between *Otomops* identifications based on molecular analysis, morphometric analysis, and other means in mapping their distributions (Fig. 2). Because genetic analysis of historic specimens can be both costly and destructive, finding qualitative morphological characters that will allow robust diagnosis should be a priority. Our samples of the two species appear to differ in size as described by Ralph et al. (2015), with *O. harrisoni* averaging larger in both external and cranial morphometrics (Supplemental Information 7).

Besides swelling national species richness tallies, these records also establish a large area of potential geographic range overlap between *O. harrisoni* and *O. martiensseni*, including most of Kenya and Uganda and parts of Rwanda and Tanzania. Although our genetic sampling is limited, sympatry of the two species is unaccompanied by evidence of introgression or hybridization (see also Ralph et al., 2015). We documented both species roosting in the same cave (Musanze) on 4 October 2015. Lack of hybridization despite geographic overlap constitutes “the test of sympatry” for the application of the biological species concept (Mayr, 1963). However, the rapid flight and possibility of long-distance movements of these bats may carry them far beyond the limits of their breeding ranges (Fenton et al., 2002; Kingdon, 1974; Kock et al., 2005). Evidence for reproductive isolation between these species of *Otomops* would be stronger with overlaps during the mating season. The interactions of these bats in the mixed-species roost at Musanze require additional investigation.

The implications of our findings for conservation of these bats are also uncertain. *O. harrisoni* is currently considered vulnerable and *O. martiensseni* is considered near threatened by the IUCN (http://www.iucnredlist.org/). Range extensions of both species would suggest possible amelioration of their respective statuses. However, the potential of these bats for long-distance movements and the absence of geographic structure in their genetics (Fig. 3) suggest that our new records came from the same parent populations as previously recorded ones. Do the various records marked “x” in Fig. 2 document different colonies of *Otomops* or do they simply reflect individual movements from the same set of populations? Better baseline population estimates and annual-cycle studies at known roosts are needed to distinguish recurring seasonal variation in population size from systemic declines that warrant conservation actions (Kock et al., 2005). Colonies *O. harrisoni* in the Chyulu Hills lava tubes contained tens to hundreds of thousands in December 2012 and October 2016, whereas those at Mt. Suswa contained a few thousands in August 2011 and January 2014. At Musanze cave in October 2015, there were a few dozen individuals of *Otomops* sp. Moreover, microsatellite variation or SNPs would increase the demographic resolution offered by gene sequences (Andriollo, Naciri & Ruedi, 2015; Speer et al., 2017).

Echolocation call analysis of hand-released individuals of *Otomops* showed that peak frequency of bats from Chyulu Hills, Kenya differed significantly from those at Mt Suswa, Kenya and at Musanze, Rwanda (Table 1). Vouchers taken from both the Chyulu and Suswa roosts included only *O. harrisoni*, but the voucher samples from Musanze represented both species. Maximum and minimum frequencies also differed between sites. The call parameters from Suswa and Musanze are similar to those measured from a single hand-released bat captured at Bungule, in Kenya’s Taita Hills (Taylor et al., 2005). Curiously, Bungule lies 190 km SSE of the Chyulu locality where the calls of *Otomops* significantly differed (Fig. 2). Call parameters for all three populations fit broadly with those recorded from *O. martiensseni* in previous studies (Adams, Bonaccorso & Winkelmann, 2015; Fenton et al., 2002) and fail to document clear species-level differences at colonies containing *O. harrisoni*. Because vocalizing bats were only identified by association, it is unknown whether differences documented here reflect specific or regional differentiation. Non-invasive genetic samples (e.g., buccal swabs or wing punches) need to be taken from molossid bats used in vocalization studies in order to confirm their specific identity (Gager et al., 2016).

The ectoparasitic arthropods of *O. harrisoni* are poorly known, in part owing to its synonymic history. Beaucournu & Kock (1996) reported the flea *Araeopsylla scitula* (Rothschild) (Ischnopsyllidae) from the Mt. Suswa and Ithundu populations, noting dramatic temporal variation in flea numbers. The five species of ectoparasites we recovered from bats at the Mt. Suswa roost all represent novel records for *Otomops.* None of these species has been previously reported from *O. martiensseni* (cf. Long, 1995; Yalden & Happold, 2013).

*Otomops harrisoni* and *O. martiensseni* represent faunal elements typical of northeastern and more southern African biotas, respectively. Their geographic replacement across Kenya, northern Tanzania, and Rwanda reinforces the region’s characterization as a transition or suture zone between biotas of the Horn of Africa and those of the savannas of Eastern and Southern Africa (Happold & Lock, 2013; Linder et al., 2012). Other species pairs showing geographic replacement in this region include Somali and Common ostrich (*Struthio molybdophanes* and *S. camelus*), Reticulated and Maasai giraffe (*Giraffa reticulata* and *G. tippelskirchi*), Beisa and Fringe-eared oryx (*Oryx beisa* and *O. callotis*), and Guenther’s and Kirk’s dikdiks (*Madoqua guentheri* and *M. kirki*). Whereas these terrestrial species show rather narrow replacement zones, these highly mobile bats exhibit a far more extensive zone of overlap, comprising nearly the entire latitudinal breadth of Kenya. Overlap of these species may be facilitated by their strong flight and very spotty known distributions.

## Conclusion

Despite modest genetic divergence, the two African species of *Otomops*, *O. martiensseni* Matschie, 1897, and *O. harrisoni* Ralph et al., 2015, appear to be valid biological species. Their ranges are shown to overlap over most of Kenya, with the southern African form ranging nearly to Ethiopia and the northeastern African and Arabian species ranging southwest into Rwanda. Both species were documented as roosting in the same cave in northern Rwanda, raising additional questions about their ecological and reproductive compatibility.

## Supplemental Information

10.7717/peerj.4864/supp-1 Supplemental Information 1 List of specimens used in genetic analyses of *Otomops*.

10.7717/peerj.4864/supp-2 Supplemental Information 2 List of specimens used in mapping the distribution of *Otomops*. Specimen details, localities, and Genbank accession numbers of sampled individuals of *Otomops*: BMNH — Natural History Museum, London; DBN—Durban genetic samples; DM — Durban Natural Science Museum; FMNH — Field Museum of Natural History, Chicago; HZM — Harrison Zoological Museum, Sevenoaks; KZN — KwaZulu-Natal genetic samples; LACM — Los Angeles County Natural History Museum; MHNG — Museum d’Histoire Naturelle, Geneva; MNHB — Museum der Naturkunde für Humboldt Universität zu Berlin; MVZ — Museum of Vertebrate Zoology; N/A — none available; NMK — National Museums of Kenya; NMK — National Museums of Kenya, Nairobi; NMP — National Museum of the Czech Republic, Prague; NMZ — Livingstone Museum; PWW — Collection of Paul W. Webala; RBINS — Royal Belgian Institute of Natural Sciences; RMAC — Royal Museum for Central Africa, Tervuren; ROM — Royal Ontario Museum, Toronto; SMF — Senckenberg Museum, Frankfurt am Main; TM — Ditsong Museum of Natural History; UMMZ — University of Michigan, Museum of Zoology; USNM — National Museum of Natural History, Washington.

10.7717/peerj.4864/supp-3 Supplemental Information 3 Alignment of mitochondrial sequences for *Otomops.*.

10.7717/peerj.4864/supp-4 Supplemental Information 4 Alignment of concatenated mitochondrial and nuclear sequences for *Otomops.*.

10.7717/peerj.4864/supp-5 Supplemental Information 5 List of vocalizations recorded from hand-released *Otomops.*.

10.7717/peerj.4864/supp-6 Supplemental Information 6 List of ectoparasites recoved from *Otomops harrisoni* at the Mt. Suswa roost.

10.7717/peerj.4864/supp-7 Supplemental Information 7 External measurements of *Otomops* specimens used in genetic analyses.

## Additional Information and Declarations

### Field Study Permissions

The following information was supplied relating to field study approvals (i.e., approving body and any reference numbers): Kenyan work was permitted by Kenya Wildlife Service, KWS/4001 and the Kenya Forest Service, RESEA/1/KFS/75). Rwandan fieldwork was carried out with authorization by the Rwanda Development Board.

### DNA Deposition

The following information was supplied regarding the deposition of DNA sequences: GenBank sequences were uploaded (MH010730–MH010797).

### Data Availability

The following information was supplied regarding data availability: The raw data are provided in the Supplemental Files.
